# Hip arthroplasty: current concepts and potential complications

**DOI:** 10.1590/0100-3984.2020.53.1e2

**Published:** 2020

**Authors:** Aline Serfaty

**Affiliations:** 1 Radiologist specializing in diagnostic imaging of the musculoskeletal system and Medical Director of the Clínica Medscanlagos, Cabo Frio, RJ, Brazil. Email: alineserfaty@gmail.com.

Total hip arthroplasty (THA) is one of the most successful surgical procedures in medical history and is an important treatment option for several end-stage hip diseases that lead to functional impairment of the joint. Annually, approximately one million patients worldwide benefit from this procedure. Although osteoarthritis is the most common indication, the procedure can also be used in the treatment of other conditions, such as femoral neck fracture, avascular necrosis of femoral head, hip dysplasia, and inflammatory arthritis^(1-3)^. 

In the mid-twentieth century, Charnley^(4)^ contributed significantly to the evolution of THA by introducing the concept of low friction arthroplasty. Since then, demand for the procedure has grown because of advances in prosthetic materials, manufacturing processes, and surgical techniques, as well as increased life expectancy^(2,3)^. Despite such progress, the implant lasts for ≤ 25 years in 58% of patients, according to a meta-analysis conducted by Evans et al.^(5)^. 

Despite the high rates of surgical success in THA, complications may occur and vary according to patient age, bone quality, and patient-specific comorbidities^(6)^. Some of the major complications are aseptic loosening, dislocation, infection, fractures, postoperative hematoma, and heterotopic ossification. Although radiography continues to be the primary diagnostic imaging method used in the short- and long-term assessment of patients with pain after THA, ultrasound, computed tomography, and magnetic resonance imaging also play important roles in this scenario. Advances in diagnostic imaging, such as conventional pulse sequence optimization and metal artifact reduction techniques in magnetic resonance imaging, can be quite useful in the detection and characterization of potential complications of THA, such as synovitis and adverse local tissue reaction^(2,3,7,8)^.For effective analysis of the images obtained in diagnostic examinations, it is essential to recognize the type of arthroplasty (hemiarthroplasty or total arthroplasty) and prosthesis (unipolar or bipolar), as well as the type of implant fixation (cemented, cementless, or with screws), because complications may originate from the presence of these materials in the hip^(1,5,7)^. 

In a pictorial essay in the current issue of **Radiologia Brasileira**, Enge Júnior et al.^(9)^ described the main complications of THA. The authors provided information on the types of arthroplasty, prostheses, and implant fixation, focusing on the main radiographic findings of the potential complications of this procedure. The authors also provided detailed characterizations of complications such as periprosthetic fracture, osteolysis/infection, wear, dislocation, and heterotopic calcification, together with illustrative figures of radiographs and CT scans. 

It is important for radiologists to be familiar with the imaging aspects of the complications of THA. The correct interpretation of these findings is crucial, not only for the outcome of each case but also for treatment planning. 
